# Benefits, for patients with late stage chronic obstructive pulmonary disease, of being cared for in specialized palliative care compared to hospital. A nationwide register study

**DOI:** 10.1186/s12904-021-00826-y

**Published:** 2021-08-24

**Authors:** Ingela Henoch, Ann Ekberg-Jansson, Claes-Göran Löfdahl, Peter Strang

**Affiliations:** 1 Department of Research and Devlopment, Angered Hospital, Gothenburg, Sweden; 2 Institute of Health and Care Sciences, Sahlgrenska Academy, University of Gothenburg, Box 457, 405 30 Gothenburg, Sweden; 3 Institute of Medicine, Sahlgrenska Academy, University of Gothenburg, Gothenburg, Sweden; 4 University of Lund, Lund, Sweden; 5 COPD Center, Institute of Medicine, Sahlgrenska University Hospital, University of Gothenburg, Gothenburg, Sweden; 6 Department of Oncology–Pathology, Karolinska Institute, Stockholm, Sweden; 7 Research and Development Unit, Stockholms Sjukhem Foundation, Stockholm, Sweden

**Keywords:** Specialized palliative care, Chronic obstructive pulmonary disease, Hospital, Symptom management, End of life care, Register study

## Abstract

**Background:**

In early stage chronic obstructive pulmonary disease (COPD), dyspnea has been reported as the main symptom; but at the end of life, patients dying from COPD have a heavy symptom burden. Still, specialist palliative care is seldom offered to patients with COPD; they more often receive end of life care in hospitals. Furthermore, symptoms, symptom relief and care activities in the last week of life for COPD patients are rarely studied. The aim of this study was to compare patient and care characteristics in late stage COPD patients treated in specialized palliative care (SPC) versus hospital.

**Methods:**

Two nationwide registers were merged, the Swedish National Airway Register (SNAR) and the Swedish Register of Palliative Care (SRPC). Patients with COPD and < 50% of predicted forced expiratory volume in 1 s (FEV_1_), who had died in inpatient or outpatient SPC (*n* = 159) or in hospital (*n* = 439), were identified. Clinical COPD characteristics were extracted from the SNAR, and end of life (EOL) care characteristics from the SRPC. Descriptive statistics were used to describe the sample and the registered care and treatments. Independent samples *t*-test, Mantel–Haenszel chi-square test and Fisher’s exact test was used to compare variables. To examine predictors of place of death, bivariate and multivariate logistic regression analyses were performed with a dependent variable with demographic and clinical variables used as independent variables.

**Results:**

The patients in hospitals were older and more likely to have heart failure or hypertension. Pain was more frequently reported and relieved in SPC than in hospitals (*p* = 0.001). Rattle, anxiety, delirium and nausea were reported at similar frequencies between the settings; but rattle, anxiety, delirium, and dyspnea were more frequently relieved in SPC (all *p* < 0.001). Compared to hospital, SPC was more often the preferred place of care (*p* < 0.001). In SPC, EOL discussions with patients and families were more frequently held than in hospital (*p* < 0.001). Heart failure increased the probability of dying in hospital while lung cancer increased the probability of dying in SPC.

**Conclusion:**

This study provides evidence for referring more COPD patients to SPC, which is more focused on symptom management and psychosocial and existential support.

## Background

Chronic obstructive pulmonary disease (COPD) is predicted to become the third leading cause of death globally by the year 2030 [1]. In its early stages, COPD is a lung disease with airway symptoms, such as shortness of breath as a main problem, especially with physical activity [2, 3]. Later in the disease trajectory, comorbidities are common, e.g. weight loss, sometimes associated with cachexia, and heart failure, resulting in increased dyspnea [4]. The risk of thromboses as well as pulmonary embolism increases, resulting in further symptoms [5, 6]. Also, depression is commonplace and is associated with poorer survival prospects [7].

At the very end of life, patients dying from COPD have similar and comparable symptoms to those dying from lung cancer [8], and are therefore in need of qualified care. For this reason, several studies comparing lung cancer and COPD have been performed [9–12]. These studies found that patients with lung cancer were more likely to receive home palliative care [9, 10] and die at home [11–13]. Specialized palliative care (SPC) was offered only in the last few weeks of life to COPD-only patients [12, 14], while COPD patients with comorbid lung cancer were far more likely to receive palliative care earlier in the disease trajectory [9]. Patients who did not receive palliative care during the last 3 months of life were more likely to die in an acute care setting [10]. Higginson et al. [15] who followed patients with COPD and interstitial pulmonary disease from 2001 to 2014, found a high prevalence of hospital deaths in both diseases, but presence of comorbidities increased the probability to die in hospital. Research in patients with advanced COPD disease has shown beneficial effects of home palliative care services, compared to usual care, on reducing symptom burden for patients [13].

Specialized palliative care focuses on symptom control, as well as on psychosocial and existential support, which includes end of life (EOL) discussions about future planning, goals of care, optimal (but not maximal) care and aims to support family well-being. In other words, the focus is on the individual patient’s wellbeing. In Sweden, most palliative care patients are enrolled in advanced palliative home care that operates on a 24/7 basis and is provided by multi-professional teams, typically including physicians, nurses, and allied health professionals. In Sweden, palliative incare services, with similar staffing, constitute an alternative for dying patients who, for certain reasons, do not want to receive care in their own homes, but prefer incare services.

Dying COPD patients have severe symptoms that need to be relieved, and SPC is a viable option. However, in contrast to lung cancer patients, COPD patients are not as likely to die in SPC: [10, 16] a considerable percentage receive their EOL care in acute hospitals, instead.

Although much is known about palliative care for COPD and lung cancer [8, 10, 16], the last week of life is rarely characterized in respect of symptoms, symptom relief, and care activities.

The Swedish Register of Palliative Care (SRPC) is a validated, nationwide quality register for EOL care with focus on symptoms and symptom relief during the last week of life [17, 18]. It is retrospectively completed and provides important data that can be compared across settings.

## Aim

The aim of this study was to compare patients with late stage COPD who were being treated in SPC versus COPD patients receiving treatment in hospital. The following research questions were asked:

- What are the demographic and clinical characteristics of patients with COPD and < 50% of predicted FEV_1_ receiving SPC, compared to hospital care?
- What characterizes the care, including symptom relief, EOL discussions, anyone present at death, and bereavement support provided to families, provided in SPC versus that provided in hospitals to patients with COPD and < 50% of predicted FEV_1_?

## Method

This is a register study where two nationwide registers were merged, the Swedish National Airway Register (SNAR) [19] and the Swedish Register of Palliative Care (SRPC). The SNAR contains data on patients diagnosed with either COPD or asthma. Health care professionals (HCPs) in outpatient units made registrations of each patient visit. Most of the registrations were made in primary health care and only 14% of registrations were made in specialized pulmonary clinics. Registrations from the SNAR included demographic, clinical, and patient-reported data. In the present study, the last registrations for COPD patients were identified.

The SRPC, a nationwide quality register of EOL care, encourages all county councils and municipalities in Sweden to retrospectively complete a questionnaire about EOL care with focus on the last week of life. Health care professionals, registered nurses in the absolute majority of cases, at the unit where the patients had died report demographic and clinical characteristics of the patients, as well as place of death, some characteristics of the EOL care, and symptoms in the last week of life. The SRPC has been validated and has previously been described in detail [17, 18]. It has a coverage of about 60% of all deaths in the country; and some of the questions from the SRPC have been adopted by the National Board of Health and Welfare as national quality indicators for a good death in Sweden [20].

Data from the two registers were merged based on patients’ personal security number. The patients included in the present study had died between 2009 and 2016.

### Sample

Of the registered patients in the SNAR, 3,114 who had died between 2009 and 2016 were identified by the Swedish Tax authorities, which registers all deaths of Swedish citizens. Of these, patients with COPD and < 50% of predicted FEV_1_ [21], corresponding to GOLD C and D, were identified, altogether 1,382. From this population, those who had died either in SPC or in hospital were extracted from the SRPC.

### Data collection

The data collection is similar to another study made by our group where we compared patients with COPD dying in nursing home with patients dying in hospitals [14]. The demographic variables retrieved from the SNAR were age, sex, and living situation, i.e., living alone or cohabiting. As in our previous study, clinical characteristics included values for FEV_1_ (forced expiratory volume during 1 s), presented as per cent of predicted, number of exacerbations and hospitalization in the last 12 months, comorbidities, and exercise capacity, measured by the number of days per week that the patient had been physically active. Patient-reported variables included smoking habits, divided into non-smokers, ex-smokers, and still smokers. Dyspnea was measured using the modified Medical Research Council (mMRC) dyspnea scale [22], ranging from 0 to 4, where 4 indicate more severe dyspnea. Health-related quality of life (HRQoL) was measured by the Clinical COPD Questionnaire (CCQ) [23], a patient-rated questionnaire with ten items where each items are scored on a 7-point scale, from zero (0) to 6, where higher score indicate more severe impact on HRQoL. In later registrations, health status was measured by the patient-rated COPD Assessment Test (CAT) [24]. The CAT consists of eight items ranging from zero (0) to 5, where 5 indicate more severe problems. The scores are summated to obtain a single total score ranging from 0 to 40.

Variables from the SRPC concerned whether death was expected; whether the patient would have preferred the place of death; presence of anyone at time death; and whether any EOL discussion about the impending death with either the patient or the family was performed; whether the family was invited to a post-death discussion; and length of stay in the setting. Concerning length of stay, seven patients with more than 1,000 days in the setting were excluded. Descriptive data and data about items such as clinical routines, symptom prevalence, and symptom management during the last week of life were also retrieved, including presence of pressure ulcers, symptom assessments, symptom prevalence, prescribed medications, and whether the symptom was alleviated. The following breakthrough symptoms during the last week of life were registered: pain, rattle, nausea, anxiety, dyspnea, and delirium (Yes/No format). The assessment of symptom relief was made on a three-grade scale: Complete – Partial – No relief. A summary of the variables is presented in Table 1.

**Table 1:** Variables included in the study. The variables listed from SNAR were included in the bivariate logistic regression analyses. Independent variables that significantly predicted the dependent variable with *p* < 0.20 in the bivariate analyses were then entered into the multivariate stepwise logistic regression analysis with the same dependent variable

**Variables from SNAR**	
Demographic variables	Age Sex Living situation, (living alone or cohabiting)
Clinical characteristics	FEV_1_ (forced expiratory volume during 1 s) Exacerbations in the last 12 months Hospitalizations due to COPD in the last 12 months Comorbidities Exercise capacity (no of days per week that the patient had been physically active)
Patient-reported variables	Smoking habits (non-smokers, ex-smokers, and still smokers Dyspnea (modified Medical Research Council dyspnea scale, mMRC) [22] Health-related quality of life (HRQoL) measured by the Clinical COPD Questionnaire (CCQ) [23] In later registrations, health status was measured by the COPD Assessment Test (CAT) [24]
**Variables from SRPC**	
	whether death was expected whether the place of death was preferred by the patient whether anyone was present at death whether there had been any EOL discussion about the impending death with either the patient or the family whether the family was invited to participate in a post-death discussion length of stay in the setting Prevalence and relief of the following breakthrough symptoms during the last week of life were registered: Pain, rattle, nausea, anxiety, dyspnea, delirium

### Data analysis

To describe the sample and the registered care and treatments, descriptive statistics were used with mean values and standard deviations (SDs) for continuous variables, and numbers and percentages of the total sample for categorical variables. Independent samples *t*-test was used to compare continuous variables and the Mantel–Haenszel chi-square test and Fisher’s exact test were used to explore relationships between dichotomous categorical variables.

To examine predictors of place of death, bivariate logistic regression analyses were performed with a dependent variable, with SPC as place of death scored as 0 and hospital as place of death scored as 1. The following independent variables were used: age, sex, living situation, FEV_1_% predicted, number of exacerbations in the last 12 months, number of hospital admissions due to COPD in the last 12 months, exercise capacity, and smoking; as well as HRQoL measured using the CCQ or CAT; dyspnea measured by the mMRC dyspnea scale; and comorbidities. Independent variables that significantly predicted the dependent variable with *p* < 0.20 in the bivariate analyses were entered into the multivariate stepwise logistic regression analysis with the same dependent variable. A similar data analysis were made in our previous study, comparing patients with COPD dying in hospitals and in nursing homes [14].

## Results

**Fig. 1:**
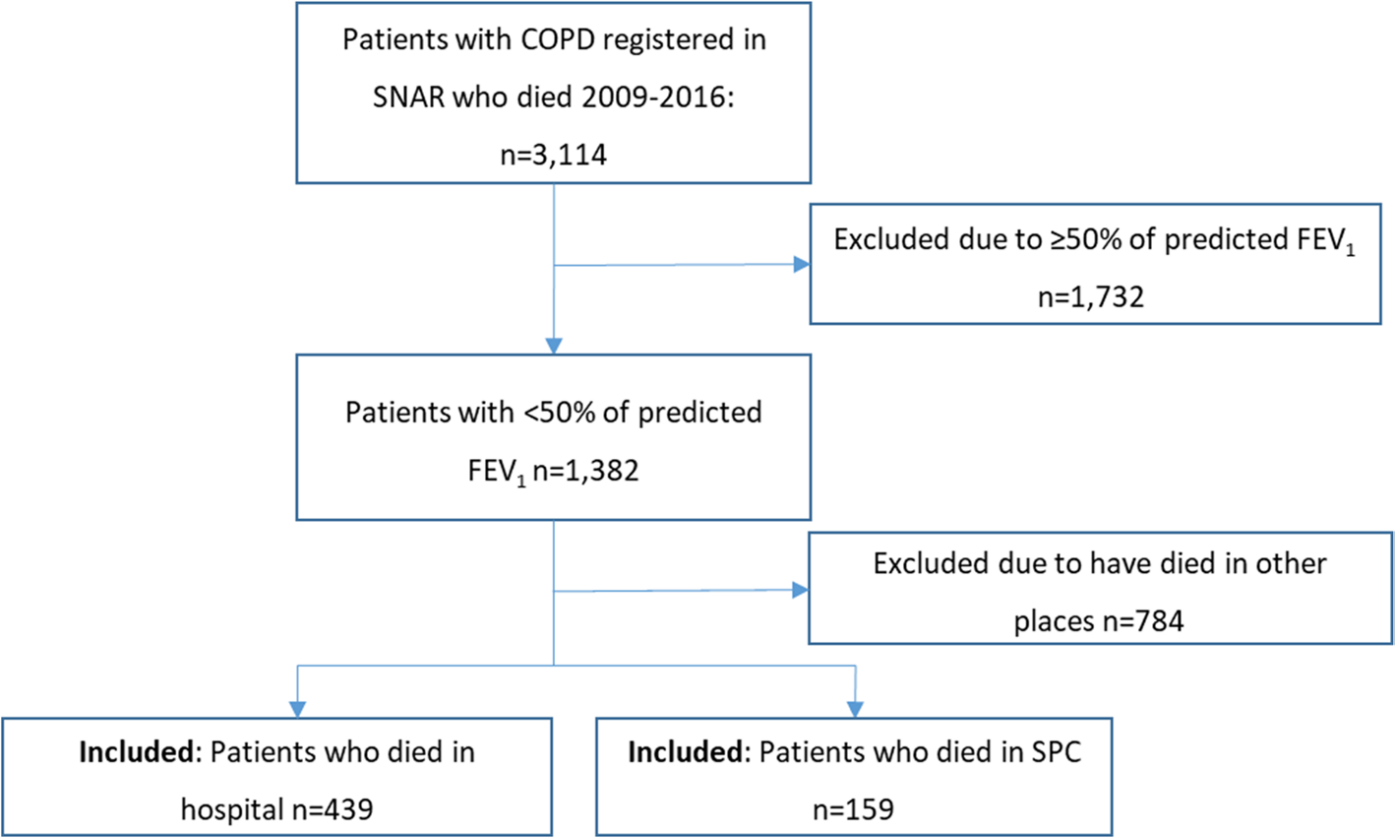
Flowchart of the patients included in the study

In total, 159 patients who had died in SPC and 439 patients who had died in hospital were identified. The patients in SPC had died either in inpatient units (*n* = 115) or in outpatient units, i.e. patients who died in their homes with support from advanced palliative home care teams (*n* = 44). A flowchart of the sample is presented in Fig. 1.

The patients who died in SPC were significantly younger than those who died in hospital. Significantly more men than women had died in SPC, while the opposite was true for those who had died in hospitals (Table 2). Length of stay in the setting was significantly longer for patients in SPC. The long time of care, 44 days was a mean value and as seen, the median value was 12 days. The much higher mean value (than median value) depended on certain patients who were enrolled in advanced palliative home care for long periods, as seen from the min–max values of 1 to 493 days. Patients who died in SPC inpatient units had shorter number of days in the setting compared to those dying at home with support from advanced palliative home care teams (mean 33 vs 81 days, *p* = 0.004, data not shown).

**Table 2:** Patients with < 50% of predicted forced expiratory volume in 1 s (FEV_1_), in both the Swedish National Airway Register (SNAR) and the Swedish Register of Palliative Care (SRPC) (*n* = 598), categorized by place of death, i.e., specialized palliative care (SPC) versus hospital. Independent samples *t*-test was used for continuous variables and Fisher’s exact test for categorical variables

	**Patients in:**		
	**Specialized palliative care (*n* = 159)**	**Hospital** **(*n* = 439)**	***P*-value, difference between SPC and hospital**
	**Mean (SD)**	**Mean (SD)**	
Time between last registered visit^a^ and death, days	682.2 (540.1)	612.2 (488.4)	0.133
Number of days in the care setting Median (min, max)	44.8 (82.6) 12 (min 1, max 493)	8.37 (9.9) 5 (min 1, max 102)	< 0.001
**Demographic variables**			
Age, years	73.5 (7.7)	75.3 (7.3)	0.008
	**n (%)**	**n (%)**	
Sex:			
Men Women	87 (54.7%) 72 (45.3%)	195 (44.4%) 244 (55.6%)	0.027
Living situation:			
Living alone Cohabiting	26 (34.2%) 50 (65.8%)	91 (47.6%) 100 (52.4%)	0.056
**Clinical variables**	**Mean (SD)**	**Mean (SD)**	
Number of exacerbations in the last 12 months	1.7 (2.4)	1.3 (1.9)	0.071
Number of hospitalizations in the last 12 months	0.7 (1.4)	0.7 (1.5)	0.71
FEV_1_% predicted	32.5 (9.3)	33.0 (9.6)	0.59
Exercise capacity (days/week)	2.0 (2.6)	2.1 (2.6)	0.84
**Patient-reported variables**	**n (%)**	**n (%)**	
Smoking:			
Non-smokers Ex-smokers Still smoking	6 (3.8%) 101 (64.3%) 50 (31.8%)	18 (4.2%) 308 (71.3%) 106 (24.5%)	0.20
	**Mean (SD)**	**Mean (SD)**	
Dyspnea (mMRC)	2.99 (1.1)	2.89 (1.1)	0.40
HRQoL (CCQ)	2.59 (1.2) (n = 84)	2.55 (1.1) (n = 182)	0.80
HRQoL (CAT)	20.4 (7.4) (n = 30)	19.95 (7.8) (n = 124)	0.77
**Comorbidity**	**n (%)**	**n (%)**	
Heart failure	16 (12.6%)	93 (28.4%)	< 0.001
Ischemic heart disease	29 (22.5%)	101 (31.1%)	0.08
Stroke	9 (8.7%)	21 (8.3%)	1.00
Hypertension	59 (43.7%)	190 (55.6%)	0.025
Diabetes	21 (15.2%)	46 (13.3%)	0.56
Osteoporosis	22 (18.2%)	70 (24.8%)	0.16
Depression/anxiety	32 (23.7%)	64 (19.4%)	0.31
Lung cancer	8 (7.4%)	7 (2.4%)	0.033
Alpha-1 antitrypsin deficiency	2 (2.0%)	4 (1.5%)	0.66

*CAT* COPD (chronic obstructive pulmonary disease) Assessment Test, *CCQ* Clinical COPD Questionnaire, *mMRC* modified Medical Research Council dyspnea scale, *SD* standard deviation

^a^ The last registered visit concerns the latest registrations that were made in the SNAR before the patients’ death

Concerning comorbidities, a greater percentage of patients dying in hospital had heart failure and hypertension, while a larger percentage of patients dying in SPC had lung cancer.

### Comparisons of clinical characteristics between settings

For findings on symptom prevalence, assessment, and management in SPC versus hospitals, see Table 3. Assessments of pain, other symptoms, and mouth health were more frequently reported in SPC than in hospitals. Although pain was more frequently reported in SPC, medication for pain was more frequently prescribed in SPC and consequently, pain was also more frequently relieved in SPC compared to hospitals. Rattle, nausea, anxiety, and delirium were reported at similar frequencies in both settings, but medication for rattle, nausea, and anxiety was more often prescribed in SPC than in hospitals. Dyspnea was more frequently reported in hospitals, but dyspnea, as well as delirium, was more frequently relieved in SPC. Pressure ulcers on admission were reported at similar levels in both settings, but pressure ulcers at death were more often reported in SPC. Parenteral infusions during the last 24 h of life were more often used in hospitals (Table 3). Patients who received an infusion had a significantly shorter length of stay in the setting, and less commonly had EOL discussions or relatives present at death, and more rarely received rescue medication for rattle and nausea (Table 4).

**Table 3:** Symptom experience, assessment, and management in specialized palliative care (SPC) and in hospitals in the total sample. Relationships between categorical variables and settings were analyzed using the Mantel–Haenszel chi-square test

	**Patients in:**		
**Palliative care characteristics**	**Specialized palliative care (*n* = 159)**	**Hospital (*n* = 439)**	***P*-value, difference between SPC and hospital**
**Symptoms and/or assessment during the last week of life**			
Assessment of symptoms other than pain	50 (35.2%)	23 (8.4%)	< 0.001
Mouth health assessment	114 (79.2%)	165 (62.5%)	0.001
Pain, prevalence	114 (73.1%)	179 (55.1%)	0.002
Pain assessment	84 (55.6%)	62 (20.7%)	< 0.001
Pain alleviated			0.001
Completely Partly Not at all	92 (80.7%) 22 (19.3%)	114 (63.7%) 62 (34.6%) 3 (1.7%)	
Prescribed rescue medication for pain	153 (97.5%)	301 (84.1%)	< 0.001
Rattle, prevalence	97 (61.8%)	203 (58.5%)	1.00
Prescribed rescue medication for rattle	152 (96.8%)	276 (77.3%)	< 0.001
Rattle alleviated			< 0.001
Completely Partly Not at all	58 (59.8%) 36 (37.1%) 3 (3.1%)	52 (25.6%) 142 (70.0%) 9 (4.4%)	
Nausea, prevalence	25 (16.2%)	46 (15.8%)	0.39
Prescribed rescue medication for nausea	143 (91.1%)	179 (51.6%)	< 0.001
Nausea alleviated			0.24
Completely Partly Not at all	17 (68.0%) 6 (24.0%) 2 (8.0%)	21 (45.7%) 22 (47.8%) 3 (6.5%)	
Anxiety, prevalence	91 (59.9%)	168 (58.3%)	0.84
Prescribed rescue medication for anxiety	154 (98.1%)	270 (77.4%)	< 0.001
Anxiety alleviated			< 0.001
Completely Partly Not at all	65 (71.4%) 26 (28.6%)	80 (47.6%) 83 (49.4%) 5 (3.0%)	
Dyspnea, prevalence	86 (56.7%)	217 (65.3%)	0.016
Dyspnea alleviated			< 0.001
Completely Partly Not at all	37 (43.0%) 48 (55.8%) 1 (1.2%)	48 (22.1%) 154 (71.0%) 15 (6.9%)	
Delirium, prevalence	45 (29.4%)	75 (26.8%)	0.91
Delirium alleviated			0.007
Completely Partly Not at all	18 (40.0%) 22 (48.9%) 5 (11.1%)	15 (20.0%) 39 (52.0%) 21 (28.0%)	
Pressure ulcer on admission			0.14
No pressure ulcer Grade 1 Grade 2 Grade 3 Grade 4	123 (82.6%) 10 (6.7%) 11 (7.4%) 3 (2.0%) 2 (1.3%)	290 (86.3%) 25 (7.4%) 14 (4.2%) 5 (1.5%) 2 (0.6%)	
Pressure ulcer at death			0.037
No pressure ulcer Grade 1 Grade 2 Grade 3 Grade 4	102 (67.5%) 22 (14.6%) 19 (12.6%) 5 (3.3%) 3 (2.0%)	260 (75.6%) 48 (14.0%) 22 (6.4%) 11 (3.2%) 3 (0.9%)	
Parenteral or enteral infusion of fluids in the last 24 h			< 0.001
No Yes	143 (95.3%) 7 (4.7%)	222 (62.5%) 133 (37.5%)

**Table 4:** Differences between patients who received an enteral or parenteral infusion of fluids and patients who did not. Relationships between categorical characteristics and infusion or no infusion of fluids were analyzed using the Mantel–Haenszel chi-square test or Fisher’s exact test

	**Patients with enteral or parenteral infusion of fluids (*n* = 140)**	**Patients with no enteral or parenteral infusion of fluids (*n* = 365)**	***P*-value**
Length of stay in the care setting, days, mean (SD)	7.2 (9.2)	44.9 (189.2)	< 0.001
	n (%)	n (%)	
End of life discussions with patient	30 (28.6%)	190 (59.4%)	< 0.001
Anyone present at time of death			< 0.001
Nobody Relative(s) Relative(s) and HCP(s) HCP(s)	29 (21.4%) 40 (29.6%) 38 (28.1%) 28 (20.7%)	79 (22.0%) 149 (41.5%) 69 (19.2%) 62 (17.3%)	
Prescribed rescue medication for rattle	103 (76.3%)	311 (85.7%)	0.036
Prescribed rescue medication for nausea	65 (49.6%)	245 (68.6%)	< 0.001

*HCP* Health care professional, *SD* Standard deviation

### Comparisons of palliative care characteristics between settings

In patients being cared for in SPC, death was more often expected; and the place of care was more often the preferred place of care, compared to those cared for in hospitals. End of life discussions with both patients and their families were more frequently held in SPC than in hospitals, and bereavement support to families was also more common in SPC (Table 5).

**Table 5:** Palliative care characteristics registered in the last week of life in specialized palliative care (SPC) and in hospitals in the total sample. Relationships between categorical characteristics and settings were analyzed using the Mantel–Haenszel chi-square test or Fisher’s exact test

	**Patients in:**		
**Palliative care characteristics**	**Specialized palliative care (*n* = 159)**	**Hospital** **(*n* = 439)**	***P*-value, difference between SPC and hospital**
Death was expected	156 (98.7%)	332 (82.8%)	< 0.001
End of life discussions with patient	123 (87.2%)	99 (34.2%)	< 0.001
Preferred place of death?			< 0.001
Yes	110 (93.2%)	23 (54.8%)	
No	8 (6.8%)	19 (45.2%)	
End of life discussion with relatives			< 0.001
Yes	134 (91.8%)	222 (72.5%)	
No	12 (8.2%)	84 (27.5%)	
Family offered bereavement support	142 (89.3%)	105 (24.2%)	< 0.001
Anyone present at time of death			0.007
Nobody Relative(s) Relative(s) and HCP(s) HCP(s)	37 (23.3%) 70 (44.0%) 32 (20.1%) 20 (12.6%)	99 (23.5%) 133 (31.6%) 90 (21.4%) 99 (23.5%)	

*HCP* health care professional

In both settings, about 23% of patients died without anyone else present. In SPC, relatives only were more often present and in hospitals, HCPs only were more often present (Table 5).

### Predictors of place of care

In the bivariate logistic regression, higher age, being a woman, living alone, having a lower number of exacerbations, having heart failure, having ischemic heart disease, and having hypertension, but not having lung cancer, predicted dying in hospital. In the multivariable stepwise logistic regression analysis, having heart failure and not having lung cancer predicted place of death, in that heart failure increased the probability of dying in hospital and lung cancer increased the probability of dying in SPC (Table 6).

**Table 6:** Logistic regression, with hospital or specialized palliative care (SPC) as place of death as dependent variable, and SPC used as reference

	**Hospital, compared to specialized palliative care, as place of death**	
	**Bivariate**	**Multivariate **^**a**^
**Independent variable**	**OR (95% CI), *p*-value**	**OR (95% CI), *p*-value**
Time between last visit and death, days	1.00 (0.99, 1.00), 0.13	
Age, years	1.03 (1.01, 1.06), 0.009	
Sex		
Men	1	
Women	1.51 (1.05, 2.18), 0.026	
Living situation:		
Living alone Cohabiting	10.57 (0.33, 0.99), 0.047	
Number of exacerbations in the last 12 months	0.91 (0.84, 1.00), 0.049	
Number of hospitalizations in the last 12 months	0.98 (0.85, 1.11), 0.71	
FEV_1_% predicted	1.01 (0.99, 1.03), 0.59	
Exercise capacity (days/week)	1.01 (0.93, 1.09), 0.84	
Smoking:		
Non-smokers	1	
Ex-smokers	1.02 (0.39, 2.63), 0.97	
Still smoking	0.70 (0.26, 1.89), 0.49	
Dyspnea (mMRC)	0.93 (0.78, 1.10), 0.40	
HRQoL (CCQ)	0.97 (0.78, 1.22), 0.80	
Heart failure	2.76 (1.55, 4.91), 0.001	3.56 (1.01, 12.51), 0.048
Ischemic heart disease	1.56 (0.97, 2.50), 0.069	
Stroke	0.95 (0.42, 2.15), 0.90	
Hypertension	1.61 (1.08, 2.41), 0.020	
Diabetes	0.85 (0.49, 1.49), 0.57	
Osteoporosis	1.49 (0.87, 2.54), 0.147	
Depression/anxiety	0.77 (0.48, 1.25), 0.30	
Lung cancer	0.31 (0.11, 0.86), 0.026	0.13 (0.02, 0.68), 0.016
Alpha-1 antitrypsin deficiency	0.74 (0.13, 4.13), 0.74	

*CCQ* Clinical COPD (chronic obstructive pulmonary disease) Questionnaire, *CI* confidence interval, *FEV*_*1*_ forced expiratory volume in 1 s; *HRQoL* health-related quality of life, *mMRC* modified Medical Research Council dyspnea scale, *OR* odds ratio

^a^All variables that significantly predicted the dependent variable with *p* < 0.20 in the bivariate analyses were entered into the multivariate stepwise logistic regression. Only significant predictors are shown in the multivariate column

## Discussion

### Patient differences

There were some significant differences in symptom prevalence and symptom relief between COPD patients in hospital and those in SPC settings, in that dyspnea was more frequently seen in hospital care and pain was more prevalent in SPC. The differences could be due to comorbidities; heart failure, which can contribute to dyspnea, was more prevalent in hospitals, well in line with a cancer study where the presence of heart failure was related to hospitalisation [25]. Lung cancer, which regularly causes pain due to metastases, was more prevalent in SPC. That pain was more prevalent in SPC settings could indicate that patients with severe pain problems more often are referred to SPC, where it is assumed that pain problems are better managed [26].

Breathlessness is a prevalent and bothersome symptom in patients with COPD, which affects functional status, distress [27], and quality of life [28, 29]. In the present study population, the prevalence of breathlessness in the last week of life was higher in hospitals. There are known differences in how patients experience breathlessness depending on which disease they have [30]. In previous research, patients with cancer described that breathlessness appeared suddenly and was frightening, while for patients with COPD, breathlessness developed gradually and was associated with episodes of distress, anxiety, panic, and fear of dying. Patients with heart failure have described the symptom in terms of limitations to daily functioning [30]. Palliative care has the potential to address breathlessness in a holistic way [30], but our study showed that patients who also suffered from diagnosed heart failure were less likely to receive palliative care, compared to patients with the comorbidity of lung cancer. This is in line with a recent review that showed that patients with lung cancer are more likely to receive palliative care compared to patients with COPD, despite a similar symptom burden [12]. However, the reason for a higher proportion of COPD patients with heart failure dying in hospitals and patients with concomitant lung cancer dying in SPC is partly explained by the nature of these comorbidities. As a rule, an acute heart failure leads to an acute hospital admission, whereas the course of a COPD patient with lung cancer is more foreseeable: a lung cancer diagnosis gives more opportunities to refer the patient to a palliative care service.

Our findings show that pressure ulcers at death were more frequent in SPC. One explanation for this could be the longer length of stay in SPC, with a longer time of being confined to bed and therefore a higher risk for pressure ulcer development. When exploring the presence of pressure ulcers in the last week of life in relation to setting, one study found that specialist inpatient palliative care units had a higher prevalence (19%) compared to hospitals (ca. 14%), when all grades of pressure ulcers were included [31]. In some cases, pressure ulcers at the end of life are unavoidable and may develop rapidly. These are often named “Kennedy Terminal Ulcers (KTUs).” Patients at the end of life have risk factors for unavoidable pressure ulcers, as they are more immobile, more malnourished, and/or cachectic.

### Patient care differences

The differences in care between SPC and hospitals were related to symptom relief, occurrence of EOL discussions, and prescription of parenteral infusions also during the last 24 h of life. Symptoms such as dyspnea, anxiety, delirium, and death rattle were more often relieved in SPC. The relieved symptoms coincide with symptom assessments, which were more frequently performed in SPC. Regular symptom assessment is associated with higher HRQoL in patients with cancer [32], and is also recommended in COPD care [33, 34].

Rescue medication was more frequently prescribed in SPC. Rescue medication has previously been found to be helpful in patients with COPD suffering from disturbing symptoms [35]. Morphine is the primary rescue medication for breathlessness in cancer patients in palliative care, but there is also evidence that morphine is helpful for COPD patients with breathlessness [36, 37].

In our population, only about one-third of patients in hospital had EOL discussions with an HCP, compared to 87% in SPC. The consequences of a lack of EOL discussions could be continued administration of unnecessary medical treatment, such as intravenous nutrition and hydration also during the last 24 h of life, which at this stage could contribute to nausea, dyspnea, and rattle. Moreover, lack of EOL discussions in the present study was also related to less prescription of rescue medication. This suggests that patients who have had EOL discussions may also receive higher quality care, possibly, as a result of higher awareness of the impending death. Previous studies report that patients with COPD were more satisfied with care after having had EOL discussions [38–40]. In the present study, higher rates of EOL discussions also coincide with higher ratings of the setting as the preferred place of death, which could be difficult for HCPs to know without bringing up the topic. Moreover, patients with lung disease and their relatives, as well as clinicians, have been reported to have a positive attitude to introducing advance care planning in a thoracic inpatients ward, especially when the focus of the discussions concerns symptom control [41].

Furthermore, in our study, patients who had parenteral infusion of fluids the last week of life had also lower rates of EOL discussions, which indicates that patients and relatives may not have been informed about the risks of nutrition and fluid in the acutely dying patient. In the present study, parenteral nutrition support was more common in hospitals than in SPC. To provide COPD patients with nutritional support is important in the early stages of the disease, but, at the end of life, total parenteral nutrition could cause nausea, due to an autonomic dysfunction in the dying, resulting in a gastric distension, but also in dyspnea and rattle, due to hyperhydration [42]. Moreover, in cases when HCPs do not initiate EOL discussions and nutritional support continues to be provided, this could signal to patients and their relatives that the patient is not immediately dying.

In the present study, there were similar levels, about 23%, of patients dying alone in both settings. A study comparing deaths of patients with cancer and patients with heart failure found that 20% of patients with heart failure, compared to 12% of patients with cancer, were alone at the moment of death [43]. Furthermore, another study that explored several aspects of palliative care in patients dying in nursing homes, found that about 16% died without anyone present [44]. This could indicate that patients with COPD are more often alone at the very moment of death, even in cases where death is expected within days. Dying alone is sometimes regarded as a failure of the HCP, but can happen when death occurs suddenly, unexpectedly, or during sleep. This is a topic that needs to be communicated with relatives and in health care teams, in order to reduce feelings of guilt for not providing optimal care.

Patients might be inclined to seek care in hospitals because of the high medical competence related to hospital care. In contrast to cancer, COPD is often regarded, by both the patient and HCPs, as a “chronic disease,” and is less often viewed as a palliative diagnosis, despite high mortality. This could be due to the unpredictability of the COPD disease trajectory, especially in combination with heart failure. When presenting with an exacerbation, neither the patient nor the HCPs know whether this exacerbation is the last one leading to death. Although it was significantly more common that death was expected in SPC, still 82.8% of deaths in hospitals were expected. This could indicate that there is reason to offer SPC earlier in the disease course, and more frequently.

### Implications

In line with our results showing that breathlessness was relieved to a larger extent in SPC, early integration of palliative care with respiratory primary care and rehabilitation services has been associated with better management of dyspnea in patients with COPD [45]. Our study indicate that patients with COPD need support to manage severe symptoms including anxiety [46] and need both medical treatment and psychological support [26], which is provided in palliative care. Admissions to SPC should be considered more often, as recent Swedish data show that COPD patients admitted to SPC have a reduced need of emergency room visits and have more seldom hospital as their place of death [47]. A pre-emptive approach, instead of reacting when a high-intensity symptom already is present, is a main issue in palliative care, which is also applicable to hospital care of COPD patients. To be able to detect symptoms early, regular symptom assessment is an important prerequisite for the improvement of symptom management in all settings.

### Strengths and limitations

Strengths of this study are that breakthrough of symptoms and the degree of relief were registered systematically with a validated questionnaire, where several of the questions are among those adopted by the National Board of Health and Welfare as national quality indicators for good care of the dying [20]. Using SRPC data, the prevalence of symptoms as well as symptom relief can be compared in different settings. We have no possibility to evaluate any differences between registered patients in the SNAR and not registered. However, with the great number of patients from almost all parts of the country, we feel confident that we get representative number of patients included in this study. In SRPC, 60% of the patients who die in hospitals and 90% of the patients in SPC were registered. If a clinic is committed to register, then most of the patients in that clinic will be registered, which increases the credibility of the study.

A weakness of our study is the observational design, without any random assignment to the care settings, e.g. COPD patients with acute heart failure are often admitted to hospitals. Some of them will recover whereas others will die. Future studies should address this type of different outcomes. Other limitations are that the data were collected by HCP retrospectively and that the specialty of the hospital wards was not registered. The comparison of two different registers could not fully exclude the risk that the symptom reporting habits differ between palliative care and hospital care. Although many initial factors to compare the two groups are similar, others vary considerably. Most importantly, the comorbidity spectrum is different between the groups, as, e.g., the hospital group more commonly had heart failure. The pace of disease progression can also be a factor that differs between the groups, influencing the selection of patients for the two care settings as emergency hospitals are equipped for emergency care, but not for planned palliative care. The mean days between the last visits registered in SNAR were 682 vs 612 (NS), indicating that the patients in the meantime probably have had health care contacts that were not registered in the SNAR.

## Conclusion

The results from this study, examining the characteristics of end of life care for COPD patients in hospital versus specialized palliative care, indicate that: (1) symptoms are prevalent in both settings, but symptom relief is offered more often in specialized palliative care than in a hospital setting; and (2) end of life communication is more common in specialized palliative care. Based on the careful registration of items importantly related to the EOL treatment of patients with COPD, and in spite of the abovementioned limitations of this observational study, our findings indicate that referring COPD patients to specialized palliative care needs to be considered. An important option for that care is an outpatient setting, which can also be viewed as a transitional phase from hospital care.

## Data Availability

The datasets analysed during the current study are available from the corresponding author on reasonable request. As regards the primary databases used in this study, SNAR is found in the SNAR’s website [48] and SRPC is found in SRPC’s website [49]. SRPC is partly open, aggretated data are available from their website [49]. However, for individual data, an administrative permission is needed for both registers, which was received for SNAR 2015–08-01 and for SRPC 2017–03-28.

## Abbreviations

CAT: COPD Assessment Test
CCQ: Clinical COPD Questionnaire
COPD: Chronic obstructive pulmonary disease
EOL: End of life
FEV_1_%: Per cent of predicted forced expiratory volume in 1 s
HCP: Health care professionals
mMRC: Modified Medical Research Council
SNAR: Swedish National Airway Register
SPC: Specialized palliative care
SRPC: Swedish Register of Palliative Care
